# Performance and carcass characteristics of Nellore cattle fed a high-concentrate diet with a phytogenic additive blend

**DOI:** 10.1371/journal.pone.0335189

**Published:** 2025-10-16

**Authors:** Jéssica Geralda Ferracini, Rodrigo José de Oliveira, Mariana Bassanezi Gasparim, Daniel Polli, Luanda Torquato Feba, Gercino Ferreira Virginio Júnior, Ivanor Nunes do Prado, Danilo Domingues Millen

**Affiliations:** 1 Department of Animal Science, State University of Maringá, Maringá, Paraná, Brazil; 2 São Paulo State University (UNESP), School of Agricultural and Veterinary Sciences, Jaboticabal, São Paulo, Brazil; 3 Department of Agronomy, University of Western São Paulo, Presidente Prudente, São Paulo, Brazil; 4 São Paulo State University (UNESP), School of Agricultural and Technological Sciences, Dracena, São Paulo, Brazil; University of Guelph Ontario Agricultural College, CANADA

## Abstract

This study evaluated the effects of a phytogenic feed additive – a blend of tannins, flavonoids, and essential oils – on intake, performance, and carcass traits of feedlot-finished Nellore cattle. The additive was hypothesized to enhance performance by modulating ruminal fermentation, favoring more efficient energy utilization pathways. Ninety-six intact Nellore bulls (357.4 ± 25.9 kg) were assigned to 24 pens (4 animals/pen) and fed high-concentrate diets for 116 days. Treatments included: 1) control diet and 2) control plus the phytogenic additive blend. Cattle receiving the phytogenic blend showed a significant reduction in dry matter intake (DMI) as a percentage of body weight (P = 0.04) without changes in final body weight, average daily gain, or carcass ultrasound traits. A numerical improvement in feed efficiency (3.3%; P = 0.19) was observed, along with increased selection for long particles (P = 0.02), potentially indicating altered feeding behavior. No differences were detected in fecal starch content or total starch digestibility. While the phytogenic blend did not significantly enhance performance or carcass traits, the reduction in intake without impairing productivity suggests a potential improvement in feed utilization efficiency. Further research is needed to refine dosing strategies and evaluate their effects under varying feeding conditions.

## Introduction

Rumen microbial fermentation plays a central role in meeting the energy requirements of ruminants by producing short-chain fatty acids (SCFAs), such as acetate, propionate and butyrate, which are then absorbed by the animal [1]. However, high-concentrate diets rich in rapidly fermentable carbohydrates can lead to excessive SCFA accumulation, which lowers ruminal pH and increases the risk of subacute ruminal acidosis (SARA), thereby impairing nutrient digestion and feed efficiency [2,3]. Reduced chewing activity and salivary buffering in low-fiber diets further exacerbate these risks [4].

Feed additives are widely used in intensive production systems to mitigate such challenges and improve performance [5]. Ionophores, especially monensin, are effective in modulating fermentation by selectively inhibiting gram-positive bacteria, reduce methane production, and favoring propionate synthesis [6]. However, concerns related to antimicrobial resistance and residues in animal products have increased the demand for natural alternatives, particularly in markets where ionophores are restricted or banned [7].

Phytogenic compounds such as tannins, flavonoids, and essential oils have emerged as promising alternatives due to their antimicrobial, antioxidant, and fermentation-modulating effects [7,8]. Previous studies report variable outcomes, ranging from improved feed efficiency and performance [9,10] to no significant differences [11,12], which may reflect differences in diet composition, inclusion rates, or interactions among bioactive molecules [13].

Despite the growing use of phytogenic blends, evidence regarding their effects in high-concentrate diets for *Bos indicus* cattle is still limited. Based on this context, we hypothesized that supplementation with a phytogenic additive blend composed of tannins, flavonoids, and essential oils would improve feedlot cattle performance, potentially enhancing feed efficiency. Therefore, the objective of this study was to evaluate the effects of a phytogenic blend on feed intake, growth performance, and ultrasound-based carcass traits of Nellore bulls finished on high-concentrate diets.

## Materials and methods

Animal care and handling used in this experiment adhered to the guidelines of the Animal Use Ethics Committee (CEUA) and were approved by the Ethics Committee on Animal Use of São Paulo State University (UNESP), Dracena campus Brazil (Protocol CEUA 021/2022).

### Animals, experimental design, and diets

The study was conducted at a commercial feedlot (Gasparim Sementes e Nutrição Animal, Presidente Bernardes, São Paulo, Brazil) using a randomized complete block design. A total of 96 non-castrated Nellore bulls (357.4 ± 25.9 kg initial body weight, BW; 22 months old) were allocated to 24 pens (4 animals per pen; 0.62 m of linear bunk space and 24 m² of pen area per animal). Pens were blocked by initial BW, and each pen was considered the experimental unit.

Cattle were fed *ad libitum* twice daily (0900 and 1400 h) with free access to water. Experimental diets were formulated to be isonitrogenous and isoenergetic using the Large Ruminant Nutrition System (LRNS; [14]; Table 1). Diets were fed in three phases: adaptation (days 1–14), growing (days 15–56), and finishing (days 57–116). The only difference between treatments was the inclusion of the phytogenic additive, a blend containing sepiolite, chestnut extract, benzoic acid, geranyl acetate, essential oil extract, polyphenols, linalool, eugenol, and propyl gallate. The two experimental treatments were: 1) Control: Basal diet with no additive; 2) Phytogenic: Basal diet plus 168 ppm of the phytogenic additive.

**Table 1:** Ingredient and nutrient composition of the experimental diets.

Ingredients, % DM	Adaptation (d 1–14)	Growing (d 15–56)	Finishing (d 57–116)
Corn silage	16.0	14.0	12.0
Sugarcane bagasse	13.0	10.0	7.0
Finely-ground corn	35.0	45.0	55.0
Soybean hulls	11.3	8.2	5.1
Cottonseed meal	20.0	17.0	14.0
Protected fat	1.5	2.5	3.5
Urea	0.4	0.5	0.6
Mineral supplement ^1^	2.8	2.8	2.8
**Nutrient composition, % DM**			
Dry matter	64.0	67.0	70.0
Total digestible nutrients	67.0	69.0	73.0
Neutral detergent fiber	38.6	33.2	27.9
Crude Protein	14.4	13.8	13.3
Ether extract	3.4	4.5	5.7
Calcium	0.71	0.82	0.90
Phosphorus	0.29	0.31	0.32

^1^ Composition based on kg of dry matter: Ca 160 g, P 22 g, Na 70 g, K 40 g, Mg 35 g, S 25 g, Co 30 mg, Cu 450 mg, I 25 mg, Mn 850 mg, Se 5 mg, Zn 1350 mg, Cr 15 mg, Vitamin A 60,000 IU, Vitamin D 8,000 IU, Vitamin E 480 IU.

Feed intake was recorded daily by weighing the offered feed and orts. Dry matter intake (DMI) was expressed in kg/day and as a percentage of body weight (BW) measured at each interval. DMI variation, calculated as the absolute difference in DMI between two consecutive days, was expressed in kg and as a percentage variation [15]. Net energy for maintenance (NEm) and gain (NEg) were estimated according to Lofgreen and Garrett [16], NASEM [17], and Zinn and Shen [18] and an observed-to-expected ratio was calculated based on the values generated by LRNS.

### Animal performance and ultrasound measurements

Body weights were recorded on days 0, 28, 56, 84, and 116. Cattle were fasted for 16 hours before weighing on days 0 and 116. On intermediate days, full BW was adjusted using the NASEM [17] standard factor (shrunk BW = full BW × 0.96). Average daily gain (ADG), feed efficiency, dry matter intake (DMI), and DMI variation were calculated over the intervals 0–28, 0–56, 0–84, and 0–116 days. Feed efficiency was calculated as ADG divided by DMI.

Carcass traits were assessed by ultrasound imaging on days 0 and 116 using an Aloka SSD-1100 Flexus RTU unit (Aloka Co. Ltd., Tokyo, Japan) with a 17.2 cm, 3.5 MHz linear probe. Ribeye area (REA), subcutaneous fat thickness over the 12th rib (backfat), fat thickness over the *Biceps femoris* (P8), and marbling were measured via ultrasound at the beginning and end of the study. The REA and backfat were obtained according to Perkins et al. [19], while P8 and marbling were measured according to Rigueiro et al. [20]. Daily gains in these parameters were calculated as the difference between the two measurements divided by days on feed.

### Particle sorting and fecal starch

On days 73 and 105, diet and ort samples were collected for particle size analysis using the Penn State Particle Separator (Nasco, Fort Atkinson, WI, USA) with sieves of 19 mm, 8 mm, 1.18 mm, and bottom pan [21]. A particle sorting value of 1 indicated no sorting, values <1 indicated selective refusals (sorting against), and values >1 indicated preferential consumption (sorting for).

Fecal samples were collected from one randomly selected animal per pen on days 81–83. A composite sample (~200 g) per animal was stored at –20 °C for starch analysis following Hendrix [22] and Pereira and Rossi [23]. Total tract starch digestibility was calculated based on fecal starch concentration as described previously by Zinn et al. [24].

### Slaughter

After 116 days, cattle were slaughtered at a commercial slaughterhouse (Naturafrig Alimentos, Pirapozinho, SP, Brazil) following standard industry and animal welfare procedures in accordance with Brazilian regulations [25]. No carcass data were collected due to slaughterhouse restrictions, and carcass traits were therefore evaluated only by ultrasound.

### Statistical analyses

All data were analyzed using the MIXED procedure of SAS (v. 9.1). The model included treatment as a fixed effect and block as a random effect. Pen was the experimental unit. Data were tested for normality (Shapiro–Wilk and Kolmogorov–Smirnov tests) and heterogeneity of variances (using the GROUP option). Results were considered significant at P ≤ 0.05 and discussed as trends when 0.05 < P ≤ 0.10.

## pop Results

### Feedlot performance

Performance results are shown in Table 2. The inclusion of the phytogenic blend in finishing diets reduced DMI expressed as a percentage of body weight (BW) over the 116 days (2.29% vs. 2.36%; P = 0.04). However, no significant differences were observed in absolute DMI (kg/day), final BW, or ADG across any of the feeding intervals (P > 0.10). Feed efficiency was not significantly affected by treatment (P > 0.10), although numerical differences were observed. From days 0–116, feed efficiency averaged 0.16 kg/kg for the phytogenic group and 0.15 kg/kg for the control group. The DMI variation differed between treatments during the first 56 days, both in terms of kilograms (P = 0.03) and percentage variation (P = 0.01), with greater variability observed in the phytogenic group. No significant differences were found in net energy use (observed:expected ratio) for maintenance (NEm) or gain (NEg) (P = 0.16 for both).

**Table 2:** Feedlot performance of Nellore cattle-fed diets with or without a phytogenic additive blend.

Item	Phytogenic	Control	SEM ^1^	P-value
**Body weight, kg**				
Day 0	357.60	357.25	7.170	0.170
Day 28	398.61	395.04	7.650	0.290
Day 56	433.34	432.19	6.980	0.720
Day 84	481.56	476.43	7.010	0.280
Day 116	539.70	537.94	7.060	0.760
**Dry matter intake (DMI), kg**				
0 to 28 days	8.64	8.96	0.240	0.200
0 to 56 days	9.60	9.82	0.170	0.200
0 to 84 days	9.95	10.12	0.140	0.380
0 to 116 days	10.13	10.38	0.130	0.200
**DMI, % BW**				
0 to 28 days	2.27	2.37	0.040	0.120
0 to 56 days	2.41	2.48	0.030	0.070
0 to 84 days	2.38	2.44	0.020	0.110
0 to 116 days	2.29	2.36	0.020	**0.040**
**Average daily gain, kg**				
0 to 28 days	1.46	1.35	0.070	0.250
0 to 56 days	1.35	1.34	0.040	0.780
0 to 84 days	1.48	1.42	0.040	0.300
0 to 116 days	1.57	1.56	0.030	0.800
**Feed efficiency, kg/kg**				
0 to 28 days	0.17	0.15	0.008	0.130
0 to 56 days	0.14	0.14	0.004	0.360
0 to 84 days	0.15	0.14	0.003	0.090
0 to 116 days	0.16	0.15	0.003	0.190
**DMI variation, kg**				
0 to 28 days	0.60	0.54	0.050	0.290
0 to 56 days	0.75	0.67	0.040	**0.030**
0 to 84 days	0.83	0.86	0.040	0.630
0 to 116 days	0.89	0.88	0.030	0.790
**DMI variation, %**				
0 to 28 days	7.35	6.46	0.670	0.230
0 to 56 days	8.26	7.16	0.440	**0.010**
0 to 84 days	8.82	8.87	0.470	0.940
0 to 116 days	9.17	8.80	0.390	0.500
**Net energy – observed/expected** ^**2**^				
Maintenance (NEm)	1.230	1.200	0.020	0.160
Gain (NEg)	1.300	1.260	0.020	0.160

^1^ Standard error of the mean; ^2^ Ratio was calculated based on the values generated by LRNS.

### Carcass traits

Carcass characteristics assessed via ultrasound are summarized in Table 3. No significant treatment effects were observed on the ribeye area (REA), backfat thickness over the 12th rib (BF), fat thickness over the *Biceps femoris* muscle (P8), or marbling scores after 116 days (P > 0.10). Because differences were detected in initial BF and P8 thickness (P = 0.04 for both), the initial values were included as covariates in the statistical model for the respective traits (BF or P8). No differences were observed for daily gains in fat thickness or REA between treatments (P > 0.10).

**Table 3:** Carcass ultrasound measurements of Nellore cattle-fed diets with or without a phytogenic additive blend.

Item	Phytogenic	Control	SEM ^1^	P-value
**Ribeye area (cm²)**				
Day 0	57.33	56.83	1.060	0.740
Day 116	89.75	88.86	0.760	0.240
Daily gain	0.28	0.27	0.006	0.240
**12th rib fat, mm**				
Day 0	2.46	2.32	0.050	**0.040**
Day 116	5.31	5.17	0.200	0.600
Daily gain	0.03	0.02	0.002	0.600
**P8**^**2**^ **fat thickness, mm**				
Day 0	3.64	3.31	0.110	**0.040**
Day 116	8.51	7.99	0.300	0.240
Daily gain	0.43	0.39	0.003	0.240
**Marbling (%)**				
Day 0	2.15	1.95	0.080	0.060
Day 116	2.81	2.83	0.040	0.680
Daily gain	0.76	0.79	0.040	0.680

^1^ Standard error of the mean; ^2^ *Bíceps femoral*.

### Particle sorting and fecal starch

Results for particle sorting behavior and fecal starch concentration are presented in Table 4. Cattle fed the phytogenic blend showed greater selection for long particles (sorting index: 1.04 vs. 0.96; P = 0.02). No treatment differences were observed for medium, short, or fine particles (P > 0.10). Fecal starch concentration and total tract starch digestibility were not affected by treatment (P > 0.10). Mean fecal starch content was 17.15% in the phytogenic group and 15.91% in the control group, with total tract digestibility of 88.63% and 89.44%, respectively.

**Table 4:** Particle sorting behavior and fecal starch content of Nellore cattle-fed diets with or without a phytogenic additive blend.

Item	Phytogenic	Control	SEM ^1^	P-value
**Particle sorting**				
Long	1.04	0.96	0.021	**0.020**
Medium	1.03	1.03	0.007	0.970
Short	1.00	1.01	0.002	0.400
Fine	0.99	0.99	0.002	0.680
**Starch (%)**				
Feces	17.15	15.91	1.690	0.600
Total tract digestibility	88.63	89.44	1.370	0.680

^1^ Standard error of the mean.

## Discussion

The inclusion of a phytogenic additive composed of tannins, flavonoids, and essential oils in high-concentrate diets for feedlot-finished Nellore cattle led to a reduction in DMI expressed as a percentage of body weight, without compromising body weight gain, feed efficiency, or carcass characteristics. These findings suggest a potential improvement in nutrient utilization efficiency, even in the absence of significant differences in productive performance metrics.

It is important to note that the observed effects of the phytogenic additive may depend strongly on its specific composition. In this study, the additive was a complex blend of tannins, flavonoids, and essential oils, whereas previous studies often used single-compound products or simpler formulations. Variations in the type and concentration of bioactive compounds can influence ruminal microbial modulation, fermentation patterns, and animal responses, highlighting that comparisons across studies should consider the additive’s chemical profile and dosage [8,26,27]. Therefore, the responses reported here may not be generalizable to all phytogenic products, and the formulation must be carefully matched to the diet type and animal category to achieve consistent effects.

The reduction in DMI may reflect physiological effects associated with the bioactive components of the additive. Tannins and essential oils are known to alter the rumen microbial ecosystem, particularly by inhibiting the growth of gram-positive bacteria and protozoa, which in turn can decrease methane production and shift fermentation toward more glucogenic volatile fatty acids, such as propionate [8,28]. This fermentation profile is energetically more efficient, supporting the hypothesis that phytogenics can enhance energy yield per unit of feed consumed.

Although no differences in ADG or feed efficiency (FE) were observed, the numerical improvement in FE (3.3%) during the whole period (0–116 days) and the maintenance of performance despite reduced intake suggest a degree of compensation, likely through improved ruminal fermentation or digestion kinetics. Similar patterns were observed by Rossi et al. [10], who reported enhanced propionate proportions and reduced DMI in dairy cows supplemented with a similar blend of tannins, flavonoids, and essential oils. In their study, cows received 10 g/head/day of a powder containing 10% of the blend, corresponding to 1 g of active compounds per animal per day. In our study, cows were fed the basal diet plus 168 ppm of the phytogenic additive, representing a comparable inclusion of active compounds on a dietary basis. This similarity in dosage may help explain the comparable effects on propionate proportions and feed efficiency. Differences in diet composition, animal type, or experimental conditions could also contribute to any variations observed between studies. On the other hand, the lack of significant improvements in carcass traits aligns with the findings of Wang et al. [11], who showed no performance benefits in beef cattle supplemented with phytogenic compounds under high-energy diets.

The greater selection for long particles observed in the phytogenic group may indicate altered feeding behavior induced by the phytogenic blend. Long particle selection is typically associated with increased chewing and rumination activity, which enhances saliva production and improves rumen buffering capacity [29]. This behavioral response could serve as a compensatory mechanism to maintain rumen pH, particularly when exposed to dietary compounds that may alter fermentation dynamics or microbial activity.

Despite the observed behavioral adaptation, no significant differences were found in fecal starch content or total tract starch digestibility between treatments. This suggests that the additive did not alter starch digestion efficiency in the rumen or post-ruminal compartments. The similarity in digestibility may help explain the absence of performance differences, as energy availability from starch remained unchanged. In contrast, previous studies have reported changes in nutrient digestibility with phytogenic compounds. For example, supplementation with *Ferulago angulata* essential oil (FAE) in fattening lambs decreased dry matter intake and nutrient digestibility (CP, fiber and EE) at higher inclusion levels (up to 750 mg/kg DM), while lower doses had smaller or negligible effects [30]. These discrepancies may be attributed to differences in basal diet composition, additive dosage, or duration of the feeding period, highlighting the importance of dosage and dietary context when evaluating the effects of phytogenic additives on nutrient utilization.

During the early feeding phase (d 0–56), DMI variation was greater in the phytogenic-supplemented group than in the control group, which may reflect a transient adaptation to the diet or other environmental factors. Although high intake variability is typically associated with reduced performance and a greater risk of ruminal acidosis [31], the variation observed in this study remained below the 10% threshold considered detrimental, and no adverse effects on performance were detected.

The absence of improvement in net energy use (observed:expected NEm and NEg) and the lack of additive effects on carcass ultrasound traits highlight the importance of matching phytogenic additive formulation and dosage to specific diet types and animal categories. We also note that marbling measurements in non-castrated Nellore bulls are inherently limited due to naturally low intramuscular fat levels. Therefore, ultrasound-based marbling should be interpreted descriptively and with caution. It is possible that the 168-ppm inclusion level was insufficient to elicit a more robust productive response in Nellore bulls fed high-energy diets or that the rumen adaptation phase to the additive requires further optimization.

Overall, these findings indicate that while the phytogenic blend used in this study did not enhance growth performance or carcass traits, its capacity to reduce dry matter intake relative to body weight without impairing productivity suggests a potential improvement in feed efficiency. Although feed efficiency and starch digestibility were not statistically improved, the intake reduction reflects a more efficient use of feed for maintaining growth.

## Conclusions

Inclusion of a phytogenic additive blend composed of tannins, flavonoids, and essential oils in high-concentrate diets for feedlot-finished Nellore cattle reduced dry matter intake relative to body weight without compromising growth performance or carcass characteristics. Although no statistically significant improvements were observed in feed efficiency or starch digestibility, the intake reduction indicates a potential positive effect on feed utilization. Further research is warranted to explore dose-response effects, different phytogenic combinations, longer feeding periods, and detailed evaluation of feed intake patterns to better understand the mechanisms involved and optimize additive use under different dietary and animal management conditions.
